# Maternal and neonatal factors associated with mode of delivery under a universal newborn hearing screening programme in Lagos, Nigeria

**DOI:** 10.1186/1471-2393-9-41

**Published:** 2009-09-04

**Authors:** Bolajoko O Olusanya, Olumuyiwa A Solanke

**Affiliations:** 1 Maternal and Child Health Unit, Department of Community Health and Primary Care College of Medicine, University of Lagos, Surulere, Lagos, Nigeria; 2 Lagos Island Maternity Hospital, Catholic Mission Street, Lagos, Nigeria

## Abstract

**Background:**

Emerging evidence from a recent pilot universal newborn hearing screening (UNHS) programme suggests that the burden of obstetric complications associated with mode of delivery is not limited to maternal and perinatal mortality but may also include outcomes that undermine optimal early childhood development of the surviving newborns. However, the potential pathways for this association have not been reported particularly in the context of a resource-poor setting. This study therefore set out to establish the pattern of delivery and the associated neonatal outcomes under a UNHS programme.

**Methods:**

A cross-sectional study in which all consenting mothers who delivered in an inner-city tertiary maternity hospital in Lagos, Nigeria from May 2005 to December 2007 were enrolled during the UNHS programme. Socio-demographic, obstetric and neonatal factors independently associated with vaginal, elective and emergency caesarean deliveries were determined using multinomial logistic regression analyses.

**Results:**

Of the 4615 mothers enrolled, 2584 (56.0%) deliveries were vaginal, 1590 (34.4%) emergency caesarean and 441 (9.6%) elective caesarean section. Maternal age, parity, social class and all obstetric factors including lack of antenatal care, maternal HIV and multiple gestations were associated with increased risk of emergency caesarean delivery compared with vaginal delivery. Only parity, lack of antenatal care and prolonged/obstructed labour were associated with increased risk of emergency compared with elective caesarean delivery. Infants delivered by vaginal method or by emergency caesarean section were more likely to be associated with the risk of sensorineural hearing loss but less likely to be associated with hyperbilirubinaemia compared with infants delivered by elective caesarean section. Emergency caesarean delivery was also associated with male gender, low five-minute Apgar scores and admission into special care baby unit compared with vaginal or elective caesarean delivery.

**Conclusions:**

The vast majority of caesarean delivery in this population occur as emergencies and are associated with socio-demographic factors as well as several obstetric complications. Mode of delivery is also associated with the risk of sensorineural hearing loss and other adverse birth outcomes that lie on the causal pathways for potential developmental deficits.

## Background

It is now widely acknowledged that effective efforts aimed at improving child health in resource-poor countries must be preceded and underpinned by improvement in maternal health within a continuum of care from pregnancy to adolescence [1]. This is corroborated by substantial evidence showing that regions such as sub-Saharan Africa and South Asia with the highest rates of maternal mortality also have the highest burden of infant and child mortality worldwide [2-5]. Maternal deaths in these regions are predominantly attributable to obstetric complications during pregnancy and childbirth [3], and can be averted or substantially curtailed through availability and access/proximity to modern obstetric services by skilled attendants [6]. In many developing countries, such services are found mostly in urban areas, but they are increasingly being directed to rural areas under various global health initiatives [7].

In many developing countries undue delays in initiating life-saving surgical intervention for women at risk of severe complications among other factors may undermine the envisaged outcomes from facility-based services [3,8]. Such delays may be due to delay in seeking essential obstetric care; in reaching the hospital or appropriate health facility; or in receiving adequate care in the hospital [8]. For example, barring the absence of other barriers such as cost and accessibility, refusal of life-saving caesarean section is not uncommon among women in urban settings in sub-Saharan Africa particularly in a country like Nigeria which is a leading contributor to regional and global burden of maternal mortality [9-13].

Typically, in secondary and tertiary maternity hospitals many mothers arrive in a moribund state or with complications that may either lead to death or severe morbidity and disability in the mother after emergency surgical intervention [3,14,15]. While perinatal mortality associated with complications during pregnancy and childbirth has been extensively reported in the literature, evidence linking mode of delivery (with or without obstetric complications) to the risk of developmental disabilities in the surviving newborns in developing countries is rare [16].

Emerging evidence from a recently concluded pilot universal newborn hearing screening (UNHS) programme in a maternity hospital in Nigeria for example suggests that mode of delivery may be associated with sensorineural hearing loss in the surviving newborns [17]. This is consistent with existing reports associating the greatest burden of developmental disabilities worldwide with countries such as Nigeria, India and Pakistan besides having the highest rates of maternal and child mortality [18]. However, the potential pathways for this association have not been reported. We hypothesised that adverse neonatal outcomes commonly associated with mode of delivery in a resource-poor country are directly associated with or lie on the causal pathways of developmental disabilities such as sensorineural hearing loss.

This study therefore set out to establish the pattern of delivery and the associated maternal factors; determine infant factors/neonatal outcomes associated with mode of delivery; as well as identify possible direct or indirect links between mode of delivery and developmental deficits in early infancy under a UNHS programme in a developing country.

## Methods

### Study design and population

This cross-sectional study was conducted in an inner-city tertiary maternity hospital in Lagos, Nigeria from May 2005 to December 2007. Lagos is the most populous city in sub-Saharan Africa and the hospital is the oldest maternity hospital in metropolitan Lagos providing specialist services to several private and public hospitals within and outside its catchment area. The hospital is owned and managed by the state government as a public health institution. All live births over the study period were eligible for enrolment into the study, excluding those who did not survive 24 hours after delivery. Newborns whose mothers were too ill to be interviewed including near-misses and those who died during childbirth were also excluded. Ethical approvals were obtained from Lagos State Health Management Board, Nigeria and University College London, UK as part of a wider UNHS pilot project [17].

### Main outcome variables

Three modes of delivery were considered as outcome measures namely: vaginal delivery, elective and emergency caesarean section. Vaginal delivery included spontaneous vertex and assisted/instrumental delivery such as breech, forceps and vacuum. Caesarean section was termed elective if the decision for the operation was made before onset of labour because the pregnancy was considered to be high-risk and/or mother was referred from ante-natal clinic. Elective caesarean section in this population was rarely based on non-medical reasons. Emergency caesarean section referred to operations prompted by a diagnosis of fetal distress, vaginal bleeding, premature rupture of membrane, antepartum haemorrhage or hypertensive conditions. The term also embraced emergency intrapartum caesarean section initiated during labour.

### Maternal factors

The factors of interest were guided by evidence from published literature [19-21] and the available data from hospital records of the participants. These included socio-demographic factors such as age, marital status, parity, ethnicity, religion, education, occupation, social class, type of residential accommodation (rented or owned) and sanitation facilities (shared or self-contained). Social classes were determined based on mother's education and father's occupation [22]. Social class I was termed as "high", II or III as "middle" and IV or V as "low". Maternal factors also included variables reflecting health-seeking behaviours such as antenatal care and traditional herbal drug use in pregnancy; obstetric indications for caesarean delivery such as hypertensive conditions (including pre-eclampsia, eclampsia and pregnancy induced hypertension), antepartum haemorrhage, cephalopelvic disproportion, premature rupture of membranes, prolonged and/or obstructed labour, malpresentation, previous caesarean delivery, fetal distress and other obstetric conditions such as maternal HIV status and multiple pregnancies.

### Infant factors/outcomes

The neonatal factors or outcomes of interest were infant's gender, gestational age, birthweight, Apgar scores at one minute and five minutes, hyperbilirubinaemia, admission into special care baby unit (SCBU) and hearing screening outcomes. SCBU admission is a helpful surrogate for a range of adverse perinatal conditions that cannot be readily ascertained in hospitals with limited diagnostic facilities. Hearing screening outcomes were based on a two-stage hearing screening protocol consisting of a first-stage screening with transient evoked otoacoustic emissions followed by a second-stage of automated auditory brainstem response test as previously reported [17]. Maternal and neonatal mortality associated with mode of delivery were not considered in this study due to incomplete data for the entire period of this study.

### Statistical Analysis

Cross-tabulation of the outcome and explanatory variables was done to provide a descriptive overview of our study population. Multinomial logistic regression model was used as it is an appropriate modelling tool where there are more than two discrete outcomes. It estimates the effect of the independent variables on the probability of a particular method of delivery. The three categories of delivery (vaginal, elective and emergency caesarean section) as specified in this study are sufficiently distinct to satisfy the assumption of independent alternatives. Unconditional univariable multinomial logistic regression analysis was first performed for each independent variable against the dependent variable (mode of delivery) to examine the unadjusted association with the three modes of delivery. The strength of association was estimated by odds ratios (OR) and the corresponding 95% confidence intervals (CI). All biologically plausible factors and those with significance (p<0.05) or borderline significance (0.10>p≥0.05) were entered into the multinomial multivariable logistic model to assess the effect of each variable independently on the mode of delivery while controlling for the potential confounding effects of covariates. There were no a priori hypotheses for interaction terms so these were not investigated. Finally, neonatal factors or outcomes significantly associated with mode of delivery were determined after adjusting for all socio-demographic and obstetric factors. Model performance was estimated with the Nagelkerke Pseudo-*R*^2 ^statistic (a measure of explained variation in the model). Missing data were managed by exclusion in all of the analyses. All statistical analyses were done with SPSS Windows version 16.0 (SPSS Inc, Chicago, IL, USA).

## Results

A total of 4615 (81.9%) consenting mothers with live births out of 5636 recorded deliveries (including perinatal deaths) at the hospital over the study period were enrolled. In all, 2584 (56.0%) mothers had vaginal delivery, 1590 (34.4%) emergency caesarean section and 441 (9.6%) elective caesarean section. Majority of the mothers were between the ages of 20-35 years, married, of the Yoruba ethnic group, had a minimum of secondary education, belonged to the middle social class and lived in rented accommodation with almost half (48.2%) having shared sanitation facilities (Table 1). Emergency caesarean section rate was highest among Yoruba tribe (69.6%), Christian mothers (65.1%), those living in rented accommodation (96%) and those in the middle social class (73.2%). More than half (54.2%) of mothers who had emergency caesarean section were primiparous. The obstetric profile of the mothers showed that about a third (34.9%) did not attend antenatal clinics for their current delivery and almost one-fifth (19.1%) reported using herbal medication during pregnancy (Table 2). A total of 1544 (33.5%) from the total study population had at least one medical indication for caesarean section and 1352 (87.6%) of this group had caesarean section. Conversely, caesarean section was medically indicated in 66.6% of all those who had such intervention. Previous caesarean section and prolonged/obstructed labour were the commonest maternal obstetric indications in the total population. Emergency caesarean section rate was highest among mothers who did not attend antenatal clinics (53.1%). About 6% of the mothers were diagnosed with HIV.

**Table 1:** Socio-demographic factors of mothers by mode of delivery

**Factors**	**Total deliveries (%)** **n = 4615**	**Vaginal delivery (%)** **n = 2584**	**Emergency caesarean section (%)** **n = 1590**	**Elective caesarean section (%)** **n = 441**
Age (Years) [a]				
< 20	77 (1.7)	41 (1.6)	32 (2.0)	4 (0.9)
20 - 35	4011 (87.1)	2275 (88.1)	1379 (87.1)	357 (81.0)
> 35	519 (11.3)	266 (10.3)	173 (10.9)	80 (18.1)
				
Marital status				
Married	4519 (97.9)	2530 (97.9)	1555 (97.8)	434 (98.4)
Not married	96 (2.1)	54 (2.1)	35 (2.2)	7 (1.6)
				
Parity				
Primiparous	2316 (50.2)	1290 (49.9)	861 (54.2)	165 (37.4)
Multiparous	2299 (49.8)	1294 (50.1)	729 (45.8)	276 (62.6)
				
Ethnicity				
Yoruba	3478 (75.4)	2055 (79.5)	1107 (69.6)	316 (71.7)
Hausa	128 (2.8)	77 (3.0)	43 (2.7)	8 (1.8)
Ibo & Others	1009 (21.9)	452 (17.5)	440 (27.7)	117 (26.5)
				
Religion				
Muslim	1888 (40.9)	1173 (45.4)	555 (34.9)	160 (36.3)
Christianity	2727 (59.1)	1411 (54.6)	1035 (65.1)	281 (63.7)
				
Education				
Primary or none	420 (9.1)	244 (9.5)	147 (9.3)	29 (6.5)
Secondary	2419 (52.4)	1344 (52.0)	872 (54.8)	203 (46.0)
Tertiary	1776 (38.5)	996 (38.5)	571 (35.9)	209 (47.4)
				
Occupation				
None	899 (19.5)	534 (20.7)	309 (19.4)	56 (12.7)
Small trade/casual job	2309 (50)	1296 (43.3)	803 (50.5)	210 (47.7)
Full-time job	1407 (30.5)	754 (29.2)	478 (30.1)	175 (39.7)
				
Social class				
High	808 (17.5)	441 (17.1)	244 (15.3)	123 (27.9)
Middle	3231 (70.0)	1791 (69.3)	1164 (73.2)	276 (62.6)
Low	576 (12.5)	352 (13.6)	182 (11.4)	42 (9.5)
				
Accommodation				
Owned	228 (4.9)	150 (5.8)	63 (4.0)	15 (3.4)
Rented	4387 (95.1)	2434 (94.2)	1527 (96.0)	426 (96.6)
				
Housing sanitation facilities				
Not Shared	2391 (51.8)	1321 (51.1)	792 (49.8)	278 (63.0)
Shared	2224 (48.2)	1263 (48.9)	798 (50.2)	163 (37.0)

Missing data: [a] = 8 (0.2%).

**Table 2:** Obstetric factors by mode of delivery

**Maternal profile**	**Total deliveries (%)** **n = 4615**	**Vaginal delivery (%)** **n = 2584**	**Emergency caesarean section (%)** **n = 1590**	**Elective caesarean section (%)** **n = 441**
Antenatal care				
One or more visits	3006 (65.1)	1910 (73.9)	745 (46.9)	351 (79.6)
None	1609 (34.9)	674 (26.1)	845 (53.1)	90 (20.4)

Herbal drug in pregnancy				
None	3732 (80.9)	2020 (78.2)	1329 (83.6)	383 (86.8)
Yes	883 (19.1)	564 (21.8)	261 (16.4)	58 (13.2)

Hypertensive conditions				
No	4317 (93.5)	2499 (96.7)	1415 (89.0)	403 (91.4)
Yes	298 (6.5)	85 (3.3)	175 (11.0)	38 (8.6)

Ante-partum haemorrhage				
No	4566 (98.9)	2582 (99.9)	1546 (97.2)	438 (99.3)
Yes	49 (1.1)	2 (0.1)	44 (2.8)	3 (0.7)

Cephalopelvic disproportion				
No	4433 (96.1)	2580 (99.8)	1443 (90.8)	410 (93.0)
Yes	182 (3.9)	4 (0.2)	147 (9.2)	31 (7.0)

Premature rupture of membranes				
No	4565 (98.9)	2568 (99.4)	1561 (98.2)	436 (98.9)
Yes	50 (1.1)	16 (0.6)	29 (1.8)	5 (1.1)

Prolonged/obstructed labour				
No	4190 (90.8)	2565 (99.3)	1205 (75.8)	420 (95.2)
Yes	425 (9.2)	19 (0.7)	385 (24.2)	21* (4.8)

Malpresentation				
No	4338 (94.0)	2521 (97.6)	1425 (89.6)	392 (89.9)
Yes	277 (6.0)	63 (2.4)	165 (10.4)	49 (11.1)

Previous caesarean section				
No	4152 (90.0)	2569 (99.4)	1325 (83.3)	258 (58.5)
Yes	463 (10.0)	15 (0.6)	265 (16.7)	183 (41.5)

Maternal HIV				
No	4350 (94.3)	2441 (94.5)	1507 (94.8)	402 (91.2)
Yes	265 (5.7)	143 (5.5)	83 (5.2)	39 (8.8)

Multiple gestations				
No	4314 (93.5)	2454 (95.0)	1458 (91.7)	402 (91.2)
Yes	301 (6.5)	130 (5.0)	132 (8.3)	39 (8.8)

Fetal distress				
No	4485 (97.2)	2581 (99.9)	1465 (92.1)	439 (99.5)
Yes	130 (2.8)	3 (0.1)	125 (7.9)	2 (0.5)

*Possible error in patient records due to prior consent for elective caesarean section at booking. However no material effect was observed when reflected in the analysis

The univariate multinomial analyses showed that apart from marital status and religion all socio-demographic and obstetric factors were significantly associated with mode of delivery. Nonetheless, all factors were entered into the multivariable logistic regression models in deriving the final prediction models. While the model consisting only of the socio-demographic factors (Table 3) explained only 6% (Nagelkerke *R*^2 ^= 0.058) of the variation in mode of delivery, the model for obstetric factors (Table 4) explained about 52% (Nagelkerke *R*^2 ^= 0.517). The addition of socio-demographic factors marginally increased the explained variations in the model for obstetric factors by 1% to about 53% (Nagelkerke *R*^2 ^= 0.534). No evidence of any collinearity among the factors was found in the final models.

**Table 3:** Socio-demographic predictors of mode of delivery after multinomial logistic regression^$^

**Factor**	**Emergency c-section vs vaginal delivery** **Adjusted odds ratio** **(95% Confidence interval)**	**Emergency c-section vs elective c-section** **Adjusted odds ratio (95% Confidence interval)**	**Vaginal delivery vs elective c-section** **Adjusted odds ratio** **(95% Confidence interval)**
Age (Years)			
< 20	1.18 (0.62 - 2.22)	1.09 (0.35 - 3.38)	0.93 (0.30 - 2.86)
20 - 35	1.0	1.0	1.0
> 35	1.58 (1.22 - 2.06)**	0.69 (0.50 - 0.95)*	0.44 (0.31 - 0.61)***

Marital status			
Married	1.17 (0.64 - 2.14)	1.61 (0.63 - 4.10)	1.38 (0.54 - 3.48)
Not married	1.0	1.0	1.0

Parity			
Primiparous	1.51 (1.26 - 1.81)***	1.27 (0.98 - 1.66)	0.84 (0.65 - 1.10)
Multiparous	1.0	1.0	1.0

Ethnicity			
Yoruba	0.76 (0.61 - 0.95)**	1.07 (0.79 - 1.45)	1.40 (1.03 - 1.91)*
Hausa	0.68 (0.40 - 1.16)	1.40 (0.60 - 3.29)	2.07 (0.87 - 4.91)
Ibo & Others	1.0	1.0	1.0

Religion			
Muslim	0.90 (0.74 - 1.09)	0.85 (0.64 - 1.13)	0.95 (0.71 - 1.26)
Christianity	1.0	1.0	1.0

Education			
Primary or none	1.15 (0.78 - 1.69)	1.27 (0.72 - 2.25)	1.10 (0.62 - 1.97)
Secondary	1.08 (0.85 - 1.39)	1.10 (0.78 - 1.57)	1.02 (0.71 - 1.46)
Tertiary	1.0	1.0	1.0

Occupation			
None	0.84 (0.66 - 1.08)	1.45 (1.00 - 2.11)*	1.72 (1.19 - 2.50)**
Small trade/casual job	0.95 (0.76 - 1.20)	0.97 (0.71 - 1.34)	1.02 (0.74 - 1.41)
Full-time job	1.0	1.0	1.0

Social class			
High	1.28 (0.86 - 1.89)	0.66 (0.38 - 1.13)	0.51 (0.30 - 0.89)*
Middle	1.37 (1.03 - 1.83)*	1.22 (0.80 - 1.85)	0.89 (0.58 - 1.35)
Low	1.0	1.0	1.0

Accommodation			
Owned	0.64 (0.43 - 0.98)*	1.33 (0.70 - 2.50)	2.06 (1.09 - 3.88)*
Rented	1.0	1.0	1.0

Housing sanitation facilities			
Not Shared	1.14 (0.94 - 1.38)	0.84 (0.64 - 1.11)	0.74 (0.56 - 0.98)*
Shared	1.0	1.0	1.0

^$^After adjusting for all obstetric factors; *p < 0.05; ** p < 0.01; ***p < 0.001. c-section = caesarean section; vs = versus/compared with

**Table 4:** Obstetric predictors of mode of delivery after multinomial logistic regression^$^

**Factor**	**Emergency c-section vs vaginal delivery** **Adjusted odds ratio** **(95% Confidence interval)**	**Emergency c-section vs elective c-section** **Adjusted odds ratio (95% Confidence interval)**	**Vaginal delivery vs elective c-section** **Adjusted odds ratio** **(95% Confidence interval)**
Antenatal care			
One or more visits	1.0	1.0	1.0
None	2.54 (2.14 - 3.03)***	3.23 (2.46 - 4.24)***	1.27 (0.96 - 1.68)

Herbal drug in pregnancy			
None	1.0	1.0	1.0
Yes	0.76 (0.60 - 0.95)*	1.06 (0.75 - 1.51)	1.40 (0.99 - 1.99)

Hypertensive conditions			
No	1.0	1.0	1.0
Yes	4.80 (3.52 - 6.52)***	1.12 (0.75 - 1.68)	0.23 (0.15 -0.36)***

Ante-partum haemorrhage			
No	1.0	1.0	1.0
Yes	72.58 (17.08 - 308.47)***	3.49 (1.04 - 11.72)*	0.05 (0.01 - 0.30)**

Cephalopelvic disproportion			
No	1.0	1.0	1.0
Yes	79.83 (29.00 - 219.78)***	1.36 (0.87 - 2.13)	0.02 (0.01 - 0.05)***

Premature rupture of membranes			
No	1.0	1.0	1.0
Yes	3.38 (1.66 - 6.87)***	1.93 (0.70 - 5.31)	0.57 (0.19 - 1.75)

Prolonged/obstructed labour			
No	1.0	1.0	1.0
Yes	59.95 (37.11 - 96.84)***	5.06 (3.16 - 8.10)***	0.08 (0.04 - 0.16)***

Malpresentation			
No	1.0	1.0	1.0
Yes	6.70 (4.82 - 9.32)***	0.91 (0.63 - 1.32)	0.14 (0.09 - 0.21)***

Previous caesarean section			
No	1.0	1.0	1.0
Yes	59.41 (34.46 - 102.42)***	0.41 (0.31 - 0.54)***	0.01 (0.00 - 0.01)***

Maternal HIV			
No	1.0	1.0	1.0
Yes	1.40 (1.00 - 1.97)*	0.45 (0.29 -0.69)***	0.32 (0.21 - 0.49)***

Multiple gestations			
No	1.0	1.0	1.0
Yes	2.39 (1.75 - 3.26)***	1.08 (0.71 - 1.64)	0.45 (0.29 - 0.70)***

Fetal distress			
No	1.0	1.0	1.0
Yes	116.25 (36.28 - 372.50)***	16.61 (4.04 - 68.29)***	0.14 (0.02 - 0.87)*

^$^After adjusting for all socio-demographic factors; *p < 0.05; ** p < 0.01; ***p < 0.001. c-section = caesarean section; vs = versus/compared with

### Emergency caesarean section compared with vaginal delivery

Older mothers, those who were first-time mothers or in the middle social class had increased odds of emergency caesarean section although 24% lower odds were observed among Yoruba mothers. As expected, all the obstetric factors increased the odds of emergency caesarean section with antepartum haemorrhage, cephalopelvic disproportion, prolonged/obstructed labour, previous caesarean section and fetal distress showing the highest odds. Although maternal HIV was associated with 40% increased odds for emergency caesarean section, the difference was only marginally significant (p = 0.054). Mothers who used herbal medications in pregnancy were found to have 24% lower odds for emergency caesarean section.

### Emergency caesarean section compared with elective caesarean section

Maternal age and occupation were the only socio-demographic factors predictive of emergency caesarean section among those who required surgical intervention. Fetal distress was associated with the largest odds for emergency caesarean section while HIV-positive status and previous caesarean section were associated with over 50% decreased odds of emergency caesarean section. Hypertensive disorders, cephalopelvic disproportion, premature rupture of membranes and malpresentation were not discriminatory among mothers who had caesarean section.

### Vaginal delivery compared with elective caesarean section

Compared to women in the active childbearing age (20 - 35 years), women older than 35 years had 56% lower odds of vaginal delivery while being a Yoruba woman or having no occupation was associated with increased odds of vaginal delivery. Similarly, living in owned residential accommodation increased the odds of vaginal delivery by two-fold compared to those in rented accommodation while expectedly, living in an apartment without shared sanitation facilities decreased the odds of vaginal delivery by 26%. Almost all the obstetric factors including multiple pregnancies but excluding premature rupture of membranes were associated with decreased odds for vaginal delivery. Lack of antenatal care and use of herbal drug in pregnancy were not significantly associated with vaginal delivery.

Overall, marital status, religion and education were not associated with any mode of delivery while maternal age was a consistent socio-demographic predictor for vaginal delivery or caesarean section.

### Neonatal outcomes/factors by mode of delivery

Neonatal factors associated with mode of delivery after adjusting for all maternal factors are presented in Table 5. Infants delivered by vaginal method or emergency caesarean section were more likely to be associated with the risk of sensorineural hearing loss but less likely to be associated with hyperbilirubinaemia compared with infants delivered by elective caesarean section. Emergency caesarean delivery was also associated with male gender, low five-minute Apgar scores and admission into special care baby unit (SCBU) compared with vaginal or elective caesarean delivery. Infants delivered by emergency caesarean section were less likely to be preterm while those delivered by vaginal method were more likely to have low birthweight compared to infants delivered by elective caesarean section.

**Table 5:** Neonatal factors associated with mode of delivery after multinomial logistic regression^$^

**Factor**	**Total (%)** **n = 4615**	**Emergency c-section vs vaginal delivery** **Adjusted odds ratio** **(95% Confidence interval)**	**Emergency c-section vs elective c-section** **Adjusted odds ratio (95% Confidence interval)**	**Vaginal delivery vs elective c-section** **Adjusted odds ratio** **(95% Confidence interval)**
Gender				
Female	2229 (48.3)	1.0	1.0	1.0
Male	2386 (51.7)	1.23 (1.03 - 1.45)*	1.04 (0.82 - 1.33)	0.85 (0.67 - 1.09)

Gestational age				
37 weeks and above	3678 (79.7)	1.0	1.0	1.0
Less than 37 weeks	937 (20.3)	0.86 (0.69 - 1.07)*	0.72 (0.53 - 0.99)*	0.85 (0.62 - 1.14)

Birthweight				
2500 g and above	4044 (87.6)	1.0	1.0	1.0
Less than 2500 g	571 (12.4)	0.79 (0.60 - 1.03)	1.36 (0.88 - 2.10)	1.73 (1.12 - 2.67)*

Apgar score at 1 minute [a]				
0 - 6	3956 (90.4)	1.15 (0.84 - 1.56)	0.82 (0.53 - 1.27)	0.72 (0.47 - 1.09)
7 - 10	419 (9.6)	1.0	1.0	1.0

Apgar score at 5 minutes [b]				
0 - 6	1244 (28.4)	1.73 (1.42 - 2.11)***	1.69 (1.27 - 2.26)*	0.98 (0.73 - 1.32)
7 - 10	3131 (71.6)	1.0	1.0	1.0

Hyperbilirubinaemia				
No	4423 (95.8)	1.0	1.0	1.0
Yes	192 (4.2)	1.39 (0.90 - 2.16)	0.42 (0.24 - 0.74)*	0.30 (0.17 - 0.55)***

Admission into SCBU				
No	4148 (89.9)	1.0	1.0	1.0
Yes	467 (10.1)	1.56 (1.16 - 2.11)**	2.19 (1.30 - 3.70)	1.40 (0.82 - 2.41)

Hearing screening tests				
Pass	4296 (93.1)	1.0	1.0	1.0
Refer	119 (2.6)	0.77 (0.46 - 1.32)	3.74 (1.10 - 12.72)*	4.83 (1.42 - 16.46)*
Incomplete	200 (4.3)	0.80 (0.52 - 1.22)	1.53 (0.73 - 3.20)	1.92 (0.92 - 4.00)

Missing data: [a], [b] = 240 (5.2%); *p < 0.05; ** p < 0.01; ***p < 0.001;

^$^After adjusting for all maternal factors as well as all covariates. SCBU = special care baby unit; C-section = caesarean section; vs = versus/compared with

## Discussion

The study has shown that the rate of caesarean section in this tertiary maternity hospital is high and the substantial proportion was by emergency surgical intervention. Our study has also shown that emergency caesarean section in this hospital is associated with a wide range of obstetric complications resulting in adverse outcomes for the surviving newborns. These findings need to be set against the backdrop of the fact that the study was conducted in an inner-city community where the vast majority of mothers belonged to the middle or high social class but over half (52%) still delivered outside hospital facilities despite access to several private and public hospitals [23]. About 57% were attended by skilled health personnel suggesting that a few mothers who delivered outside hospital were still attended by skilled health personnel. Traditional maternity homes accounted for the largest proportion (40%) of all deliveries or 77.5% of all non-hospital deliveries [23]. Our hospital is the only tertiary maternity hospital serving this population and possibly reflects the settings in many countries in sub-Saharan Africa. Evidently, maternal health-seeking behaviour in this urban setting is likely to reflect a complex interaction between socio-demographic, cultural and medical factors similar in some ways to those of mothers in rural communities [24,25].

The observed caesarean section rate in this study is comparable to other local studies [26] but higher than the reported rates from other public health institutions which are usually in the range of 5% to 43% where rates closer to the upper end are more typical in Latin America [9,14,27]. This high rate of caesarean section is not an indication of an indiscriminate preference by health professionals or mothers as has been observed in some developing countries [14,27] but of the referral status of the hospital in a community where majority of mothers prefer non-facility based delivery with a high probability of late presentation requiring surgical intervention during labour. Non-vaginal delivery is generally viewed as a sign of maternal laziness, reproductive failure or a curse from perceived enemies or deity in this population. It was therefore not uncommon even where caesarean section was indicated by past pregnancy history for women to attempt vaginal delivery until there was a glaring failure with obvious threat to the life of the mother or unborn child [28]. Qualitative studies have in fact established that some women will not even accept caesarean section under any circumstances for reasons such as the fear of pain or death, financial cost, embarrassment by friends, religious beliefs and husband's disapproval [13,25]. The delays associated with these and other factors [8,12,29] may have contributed to the high proportion of emergency caesarean section (more than three-quarters of all caesarean sections). It may be worthwhile to undertake an audit to establish the relative contributions of the various types of delays to the high rate of emergency caesarean section and cases of near-misses in this setting.

Undoubtedly, a major pathway to the high rate of emergency caesarean section in this population was the lack of antenatal care which was associated with more than two-fold risk compared to those who received antenatal care. Over half of the mothers who delivered by emergency caesarean section had no antenatal care. It was also not uncommon for some mothers who had received antenatal care to end-up with emergency caesarean section if they were culturally aversed to the potential for surgical intervention and had unsuccessfully attempted vaginal delivery within or outside a hospital setting [13,30,31]. Lack of antenatal care therefore remains a vital link between socio-demographic or obstetric risk factors and adverse pregnancy outcomes and perhaps the most modifiable of all risk factors [32].

Majority of the socio-demographic and obstetric factors associated with mode of delivery in this study accord with findings from studies in both developing and developed countries [9,14,20,21]. Factors less commonly reported are type of residential accommodation, sanitation facilities and use of herbal drug in pregnancy. These may be worth exploring more appropriately through future qualitative studies especially the use of herbal drug in pregnancy which perhaps mirrors the trend in both developed and developing countries towards the combined use of alternative/complimentary medicine and orthodox medicine to prevent adverse health outcomes or maximise treatment benefits [33].

The risks associated with obstetric complications and different modes of delivery are more commonly measured in terms of maternal and perinatal mortality [3], and this practice tends to underestimate the overall burden of the adverse health outcomes [34]. The current study provides further evidence that mode of delivery is associated with the risk of sensorineural hearing loss and complements our earlier report in which emergency caesarean section and vaginal delivery were associated with at least two-fold risk of sensorineural hearing loss in surviving newborns compared with elective caesarean section [17]. Our findings also suggest that while all caesarean sections combined may portend lesser risk for sensorineural hearing loss compared with vaginal delivery as reported by studies from countries with higher standards of obstetric practice [35,36], infants delivered by emergency caesarean section were likely to be at a greater risk than those delivered by elective caesarean section in this resource-poor setting. It is in fact common in our population for emergency caesarean section to be initiated after prolonged and unsuccessful trial of labour due to several factors including cultural aversion to surgical intervention and financial constraints. In contrast, a related study among mothers with previous caesarean section found no association between mode of delivery and sensorineural hearing loss [28] which leads us to speculate that the incidence of fetal distress and the associated risks are less likely to be pronounced in pregnancies already considered as high risk as trial of labour in this group of women is likely to be more closely monitored for caesarean section than those without such obstetric indications. Although further confirmatory tests could not be provided for the majority of infants who failed the screening tests due to a high rate of follow-up default, our two-stage screening protocol typically has a test sensitivity of 92%, specificity of 98% and positive likelihood ratio of 61 in hospital-based settings [37] and is currently the protocol of choice in many UNHS programmes worldwide.

Birth asphyxia (as indexed by low five-minute Apgar scores), hyperbirubinaemia and SCBU admission are established risk factors for a broad range of developmental disabilities such as cerebral palsy, mental retardation and specific language/attention deficit disorders [38], and the observed increased risks accord with existing extensive data on the respiratory morbidity associated with mode of delivery [26,39-41]. Other studies have also found that caesarean section is associated with adverse long-term health of survivors through reduced rates of breastfeeding [42]. While the increased risk of hyperbilirubinaemia associated with elective caesarean section warrants further investigation, the overall evidence from the current study would nonetheless suggest that the benchmark for evaluating the effectiveness of any mode of delivery should not be limited to the number of deaths averted but also the risks of developmental disabilities in surviving newborns particularly when emergency caesarean section is necessitated in already compromised infants. Mothers and health care providers need to appreciate this added dimension to obstetric care in developing countries. The association between infant's gender and mode of delivery was an unusual finding that needs to be explored further in future studies.

Major advantages of this study include the comparative analysis of three modes of delivery simultaneously together with the associated neonatal outcomes seldom found in similar studies from developing countries. This study has also demonstrated that newborn screening programmes offer unique platforms for establishing interrelationships among various determinants of maternal and neonatal outcomes besides the benefits of early detection for timely intervention. However, a number of limitations of this retrospective cross-sectional study are worth noting. It is uncertain how the findings in this study can be generalised for other tertiary health institutions within different cultural settings outside sub-Saharan Africa in view of the selection bias. Adverse outcomes considered in this study excluded maternal and perinatal mortality associated with the obstetric practices but this burden is already well documented in existing literature. The potential impact of the cost of caesarean section on maternal decision was not ascertained although this was less likely to be a major factor in this state-funded public hospital compared with fees charged by private hospitals. Data on the number of antenatal visits made or the number of previous caesarean sections among those with this history was also not considered. Prospective studies addressing these limitations as well as exploring the interaction effects of parity are necessary to support the findings in this study [19]. Analysis of time interval between admission and caesarean section (to indicate degree of urgency) and analysis of subsequent length of hospital stay (to indicate severity of outcome) as well as a study of the long-term outcomes for these "near-miss" obstetric events would be valuable.

## Conclusion

This study has shown that the rate of emergency caesarean section in a tertiary referral hospital is likely to be high in a community where a high proportion of women prefer to deliver outside hospitals. This pattern of delivery is associated with several obstetric complications and adverse neonatal outcomes which portend substantial risks of developmental deficits such as sensorineural hearing loss in the surviving newborns. Efforts aimed at improving maternal and child mortality in this and similar settings must recognise the broader dimensions of the burden of obstetric complications associated with emergency caesarean section.

## Abbreviations

**C-Section**: caesarean section; **SCBU**: special care baby unit; **UNHS**: universal newborn hearing screening.

## Competing interests

The authors declare that they have no competing interests.

## Authors' contributions

BOO designed the study protocol as part of a wider post-doctoral research project. OAS participated in the data collection, contributed to data analysis and interpretation. BOO drafted the manuscript with inputs from OAS. Both authors reviewed and approved the final manuscript. BOO is guarantor of the paper.

## Pre-publication history

The pre-publication history for this paper can be accessed here:


